# SARS-CoV-2 Transmission Dynamics in Households With Children, Los Angeles, California

**DOI:** 10.3389/fped.2021.752993

**Published:** 2022-01-05

**Authors:** Melissa Lucero Tanaka, Carolyn Jennifer Marentes Ruiz, Sanchi Malhotra, Lauren Turner, Ariana Peralta, Yesun Lee, Jaycee Jumarang, Stephanie E. Perez, Jocelyn Navarro, Jennifer Dien Bard, Aubree Gordon, E. Kaitlynn Allen, Paul G. Thomas, Pia S. Pannaraj

**Affiliations:** ^1^Division of Infectious Diseases, Children's Hospital Los Angeles, Los Angeles, CA, United States; ^2^Department of Pathology and Laboratory Medicine, Children's Hospital Los Angeles, Keck School of Medicine, University of Southern California, Los Angeles, CA, United States; ^3^Department of Epidemiology, School of Public Health, University of Michigan, Ann Arbor, MI, United States; ^4^Department of Immunology, St. Jude Children's Research Hospital, Memphis, TN, United States; ^5^Department of Pediatrics and Molecular Microbiology and Immunology, Keck School of Medicine, University of Southern California, Los Angeles, CA, United States

**Keywords:** COVID-19, SARS-CoV-2, secondary transmission, attack rate, households, children

## Abstract

**Objectives:** Studies of household transmission of severe acute respiratory syndrome coronavirus-2 (SARS-CoV-2) focused on households with children are limited. We investigated household secondary attack rate (SAR), transmission dynamics, and contributing factors in households with children.

**Materials and Methods:** In this prospective case-ascertained study in Los Angeles County, California, all households members were enrolled if ≥1 member tested positive for SARS-CoV-2 by polymerase chain reaction (PCR). Nasopharyngeal PCRs, serology, and symptom data were obtained over multiple visits.

**Results:** A total of 489 individuals in 105 households were enrolled from June to December 2020. The majority (77.3%) reported a household annual income of <$50,000, and most (92.9%) were of Hispanic/Latinx ethnicity. Children <18 years old accounted for 46.9% index cases, of whom 45.3% were asymptomatic. Household index cases were predominantly children during low community transmission and adults during the high community transmission period (χ^2^ = 7.647, *p* = 0.0036. The mean household SAR was 77.0% (95% CI: 69.4–84.6%). Child and adult index cases both efficiently transmitted SARS-CoV-2 within households [81.9%, (95% CI: 72.1–91.9%) vs. 72.4% (95% CI: 59.8–85.1%), *p* = 0.23]. Household income and pets were significantly associated with higher SAR in the multivariable analysis of household factors (*p* = 0.0013 and 0.004, respectively).

**Conclusions:** The SAR in households with children in an urban setting with a large ethnic minority population is much higher than previously described. Children play important roles as index cases. SAR was disproportionately impacted by household income. Vaccination and public health efforts need special focus on children and vulnerable communities to help mitigate SARS-CoV-2 spread.

## Introduction

Severe acute respiratory syndrome coronavirus-2 (SARS-CoV-2) has continued to spread rapidly worldwide, leading to over 4.2 million deaths globally (1). Although travel and work exposure dominated initial SARS-CoV-2 spread, household exposure quickly became the principal source of transmission (2, 3). Households are high-risk settings for the spread of SARS-CoV-2 *via* respiratory droplets, aerosols, and fomites due to sustained close contact in enclosed indoor settings (4, 5). Published studies show a household attack rate of 47% and a secondary attack rate (SAR) ranging from 6 to 53 percent among household contacts (5–14), with a mean SAR of 16.6% on a meta-analysis of 44 studies by Madewell et al. (5). Previous studies suggest that children under 18 years old are unlikely to be index cases and result in a lower attack rate, that secondary attack rates are higher in adults than children, and that asymptomatic index cases have a limited role in household transmission (5, 15–18). The number of Coronavirus disease 2019 (COVID-19) cases in children, however, has been on the rise since summer 2020, paralleling trends among adults (19).

Many household studies are limited by evaluation of only a few members of the household, focusing primarily on adults in the household, using a cross-sectional or retrospective approach, studying a short period of time, or lacking serologic data. Here we describe a prospective, case-ascertained study of household SARS-CoV-2 transmission in Los Angeles, California. This is the largest household study in North America to our knowledge, the first to report findings over multiple phases of the pandemic, and one of few studies to focus primarily on households with children in an urban setting. Los Angeles County has been a hot spot of disease burden during this pandemic, with the highest COVID-19 positivity rate and the most daily new cases per 100,000 residents nationwide for most of the winter. Understanding transmission and characteristics of urban households, especially among ethnic minority populations disproportionately affected by COVID-19, will help in the development of COVID-19 prevention and mitigation measures.

## Materials and Methods

### Study Design and Participants

Households in Los Angeles County, California were prospectively enrolled into the Household Exposure and Respiratory Virus Transmission and Immunity Study (HEARTS) if at least one household member tested positive for SARS-CoV-2 within 2 weeks prior to enrollment and at least two members living in the household agreed to participate. We recruited households of patients who tested positive for SARS-CoV-2 at Children's Hospital Los Angeles (CHLA) using a convenience recruitment strategy. These individuals had presented for testing due to COVID-19 symptoms, known exposure, or in preparation for a routine procedure or hospital admission. Recruitment fliers were also posted at CHLA and at community testing sites near the hospital. We defined a household as all people who occupy a housing unit; the household may include related family members and unrelated individuals (20).

At enrollment, a designated household representative responded to a questionnaire detailing household information including the number of household members, type of housing, number of bedrooms and bathrooms, pets, smokers, and household income in the last year. In addition, each consented household member answered an individual questionnaire that included demographic information, co-morbidities, and exposure history. Co-morbidities included pre-existing lung, heart, renal, liver, or neurologic disease, diabetes, cancer or other immunosuppression. Participants logged illness symptoms in a daily symptom diary for 28 days. Parents recorded symptoms for their children. COVID-19 associated symptoms were defined as at least one of the following: fever, chills, headache, runny nose, congestion, cough, sore throat, shortness of breath, wheeze, altered smell or taste, muscle aches, or gastrointestinal symptoms. Data was recorded in the Research Electronic Data Capture (REDCap Consortium, Vanderbilt, Tennessee, USA).

Participants presented to our drive-thru respiratory testing center for nasopharyngeal (NP) swabs performed by trained study staff every 3–7 days for up to 4 weeks or until two consecutive negative SARS-CoV-2 real-time reverse transcription polymerase chain reaction (RT-PCR) results were produced. Blood was collected at the enrollment visit and a convalescent visit after 4 weeks from resolution of the last symptomatic household member. The study was approved by the Institutional Review Board at Children's Hospital Los Angeles.

### SARS-CoV-2 Reverse Transcriptase-Polymerase Chain Reaction

We tested for SARS-Cov-2 using the CDC protocol that was approved by the Food and Drug Administration (FDA) for emergency use authorization (EUA) (21). RT-PCR was performed using primers and probes that targeted the N1, N2, and RnaseP (RNP, internal control) genes (Integrated DNA Technologies, Coralville, IW). A positive result was defined as cycle threshold (CT) value <40 for both N1 and N2. A valid result for SARS-CoV-2 detection was determined by RNP using a cut-off of CT value < 32. An inconclusive result was defined as either N1 or N2 gene detected only with RNP detection.

### Serology

Serum SARS-CoV-2 receptor binding domain (RBD) and spike IgG antibody was measured using an ELISA, as previously described (22). A positive cut-off OD_490_ value of 0.2 was used for RBD based on the published protocol and the mean of the negative control values plus 3 standard deviations (SD) from 20 blood samples collected between 2017 and 2019. IgG against the spike protein was used to confirm RBD IgG positivity.

### Definitions

The attack rate of individuals per household was defined as the number of COVID-19 positive cases during the testing time period divided by the total number of household members. SAR in each household was defined as the number of new cases divided by the total number of at-risk members after subtracting out the index case. We used the earliest date between symptom onset and first positive RT-PCR result for each individual to determine the order of infection within a household. The index case was defined as the individual with the earliest onset date for symptoms or positive test in the household. If two members had the same earliest date for symptoms or positive test, they were considered co-index cases. If infection order could not be determined (e.g., asymptomatic case determined by acute/convalescent serology) for any household member, that household was excluded from the SAR analyses.

### Statistics

Comparisons of categorical variables were calculated using Pearson chi-square. Independent sample *T*-tests were used for comparison of continuous variables. Multiple linear regression analysis using the forward selection method was used to determine predictors of household attack rate and SAR. Variables were checked for multicollinearity; if found, only one variable was included in the multivariable model. All remaining factors with *p* ≤ 0.10 in the univariate analysis were included in the multivariable analysis. Statistical analyses were performed using SPSS Version 27.0 (IBM Corp., Armonk, NY). All tests were 2-tailed with *p* < 0.05 considered significant.

## Results

We enrolled 105 households with 489 individuals from June 17 to December 31, 2020, prior to COVID-19 vaccination roll-out to the general population. Three households were excluded from the analysis because only one member presented for PCR testing. One household was excluded because all members except for the index case had documented COVID-19 infection >3 months earlier. The 101 remaining households were included. Of those, 99 enrolled all individuals currently living in the household, two households each missed one individual, and one had two household members excluded due to prior diagnosed COVID-19 infection >3 months earlier. Therefore, we included 101 households with 477 individuals in the analysis.

Characteristics of the households are shown in Table 1. Households ranged from 2 to 11 members living together. All except five households had at least one child under 18 years of age. Almost half (46.5%) of the households had more than two persons per bedroom, meeting the definition for overcrowding based on the U.S. Department of Housing and Urban Development (20). The majority (77.3%) reported an annual household income of <$50,000 in the last year.

**Table 1:** Characteristics of households enrolled in a prospective study of SARS-CoV-2 household transmission—Los Angeles, California, USA, June–December 2020.

**Characteristic**	***N* = 101 households**
Number of members, median (IQR)	4 (3–6)
Number of adults, median (IQR)	2 (2,3)
Number of children, median (IQR)	2 (1–3)
Number of bedrooms, median (IQR)	2 (1–3)
Number of bathrooms, median (IQR)	1 (1, 2)
Number of persons per bedroom, median (IQR)	2 (1.5–3)
Type of Housing, *n* (%)	
House	51 (50.5)
Apartment	50 (49.5)
Pets, *n* (%)	42 (41.6)
Indoor pets	13 (2.7)
Outdoor pets	36 (7.5)
Smokers, *n* (%)	9 (1.9)
Household income, *n* (%)	
<$20,000	21 (20.8)
$20,000–$34,999	44 (43.6)
$35,000–$49,999	13 (12.9)
$50,000–$74,999	10 (9.9)
$75,000–$99,999	6 (5.9)
>$100,000	7 (6.9)

SARS-CoV-2 PCR testing of NP swabs in all 477 (100%) individuals and serology in 407 (85.3%) individuals were completed at the first visit. Individuals followed up for a median of three visits (IQR, 2–4) over 15 days (IQR, 7–27). Convalescent serology was obtained in 257 (53.9%) individuals. Overall, 393 (82.4%) tested positive for COVID-19 by PCR and/or serology at least one time point, six (1.3%) tested inconclusive by PCR with negative serology, and 78 (16.4%) tested negative for both at all time points. The COVID-19 positive individuals included 216 adults (55.0%) and 177 (45.0%) children. A little over half (233 [59.3%]) of the subjects who tested positive reported COVID-19 associated symptoms; 160 (72.7%) tested positive for SARS-CoV-2 after presenting with symptoms, 21 (9.2%) tested positive on the same day of illness onset, and 48 (21.0%) tested positive before developing symptoms. The majority of patients managed their symptoms at home, but two participants required hospitalization for COVID-19, and three participants were hospitalized for unrelated reasons. Fewer children were symptomatic compared with adults [91 (51.4%) vs. 142 (65.7%), *p* = 0.004].

Individual characteristics of all household participants, 113 index cases, and 214 secondary cases are shown in Table 2. Hispanic/Latinx ethnicity was associated with a higher individual SAR compared to non-Hispanic/Latinx ethnicity (80.0 vs. 47.4%, *p* = 0.002). This analysis excluded 18 households with 26 asymptomatic cases detected by positive SARS-CoV-2 serology only for whom infection order could not be determined.

**Table 2:** Characteristics of all enrolled subjects, index cases, and secondary cases and individual secondary attack rate of laboratory-confirmed SARS-CoV-2 infections among those at risk by characteristics, in household members enrolled in a prospective study of SARS-CoV-2 household transmission—Los Angeles, California, June–December 2020.

	**Enrolled subjects *n* = 477 (%)**	Subjects after exclusion^*^n = 382 (%)	**Index cases *n* = 113 (%)**	**Secondary cases *n* = 214 (%)**	**Secondary attack rate (%) by characteristic**	***p*-value**
**Age Group**						
<12	150 (31.4)	116 (30.4)	39 (34.5)	64 (30.6)	83.1	0.42
12–17	62 (12.9)	49 (12.8)	14 (12.4)	24 (11.5)	68.6	
18–29	97 (20.3)	82 (21.5)	21 (18.6)	49 (22.9)	80.3	
30–54	142 (29.8)	117 (30.6)	34 (30.1)	62 (29.7)	74.7	
≥55	26 (5.5)	18 (4.7)	5 (4.4)	10 (4.7)	76.9	
**Sex**						0.064
Male	220 (46.1)	179 (46.9)	51 (45.1)	93 (44.5)	72.7	
Female	257 (53.9)	203 (53.1)	62 (54.9)	116 (55.5)	82.3	
**Ethnicity**						0.002
Hispanic/Latinx	443 (92.9)	354 (92.7)	104 (92.0)	200 (95.7)	80.0	
Non-Hispanic	34 (7.1)	28 (7.3)	9 (8.0)	9 (4.3)	47.4	
/Latinx						
**Ethnicity/Race**						0.16
White	461 (96.6)	368 (96.3)	107 (94.7)	204 (97.6)	78.2	
Asian	13 (2.7)	11 (2.9)	4 (3.5)	4 (1.9)	57.1	
Multiple	3 (0.6)	3 (0.8)	2 (1.8)	1 (0.5)	100	
**Co-morbidities**						0.38
Yes	80 (16.8)	71 (18.6)	19 (16.8)	42 (20.1)	84.0	
No	397 (83.2)	311 (81.4)	94 (83.2)	167 (79.9)	78.4	
**Symptoms**						
Yes	256 (53.7)	229 (59.9)	92 (69.9)	133 (63.6)	n/a	n/a
No	221 (46.3)	153 (40.1)	34 (30.1)	76 (36.4)	n/a	n/a

* *This analysis includes 382 individuals from 83 households and excludes 18 households containing one or more case(s) for whom infection order is unknown*.

*n/a, not applicable*.

**Figure 1:**
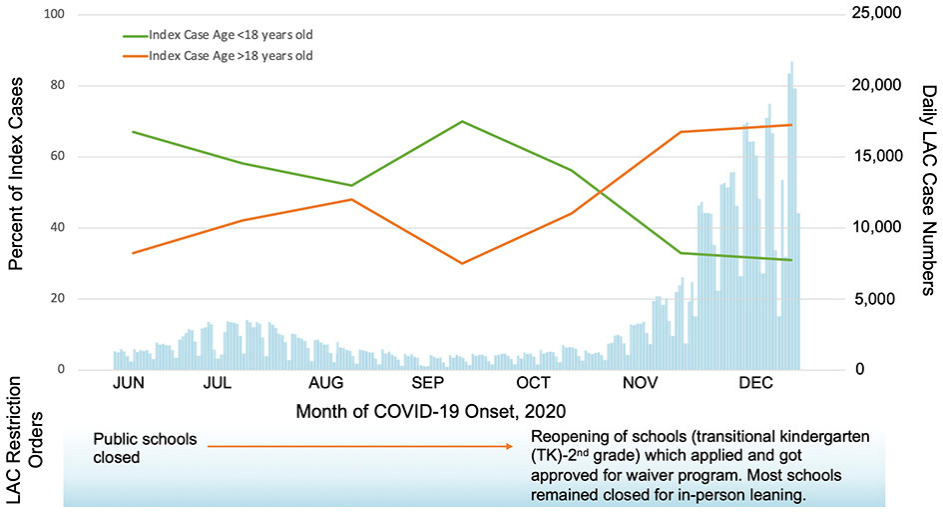
Laboratory-confirmed index cases by age group in participating households compared to Los Angeles County (LAC) daily case numbers, June-December 2020 (23). Household index cases were predominantly children during low community transmission (before November 1, 2020) and adults during the high community transmission period (after November 1, 2020), χ^2^ = 7.647, *p* = 0.006.

Overall, children under 18 years of age accounted for 53 (46.9%) index cases, of whom 24 (45.3%) were asymptomatic. Children index cases were associated with periods of lower community case rates while adult index cases were associated with periods of high community transmission and rapid incidence rise of COVID-19 cases (*p* = 0.006, Figure 1 and Supplementary Table 1). Substantial secondary transmission occurred whether the index patient was an adult or a child [81.9%, (95% CI: 72.1–91.9%) vs. 72.4% (95% CI: 59.8–85.1%), *p* = 0.23]. Symptomatic and asymptomatic index cases both transmitted SARS-CoV-2 efficiently within the household [SAR: 76.7% (95% CI: 68.2–85.2%) vs. 78.2% (95% CI: 59.3–97.2%), *p* = 0.87]. Individuals reported last face-to-face contact with confirmed COVID-19 positive household members a median of 0 days (IQR, 0–0) prior to the day of study enrollment, i.e. on the same day, suggesting very close contact in the household.

Of the 101 households, 94 (93.1%) had at least one secondary laboratory-confirmed SARS-CoV-2 infection among household members. All members tested positive in 61 (60.4%) households. The mean household SAR was 77.0% (95% CI: 69.4–84.6%) when looking at PCR and serology data together, after excluding those households with any individuals with unknown infection order. If the SAR per household was calculated solely by PCR data, it would be underestimated at 63.2% (95% CI: 55.3–71.2). Because of the risk of misclassification of asymptomatic cases and varying incubation periods (24), we performed additional analyses after reclassifying cases within 1–5 days of the index case as a sensitivity analysis (Supplementary Table 2). The SAR remained high above 63%, even with reclassification. In a univariate analysis including all households, income, ethnicity, having multiple index cases, and pets were possible predictors (*p* ≤ 0.10) of a higher household SAR. In the multivariable analysis, only household income and pets remained significant factors associated with higher household SAR (Table 3). The mean attack rate in all households was 83.9 (95% CI: 79.4–88.3%). The number of bedrooms, bathrooms, household income, multiple index cases, and Hispanic/Latinx ethnicity were each associated with a higher attack rate in univariate analyses (Table 4). However, in the multivariable analysis after removing variables with multicollinearity, only the number of bedrooms was found to be a significant predictor of higher attack rates in the household.

**Table 3:** Univariate and multivariable linear regression analysis of predictors of the household secondary attack rate (SAR).

**Factors**	**Univariate analysis**		**Multivariable analysis**	
	**Slope (95% CI)**	***p*-value**	**Slope (95% CI)**	***p*-value**
No. of household members	0.055 (-0.030 – 0.050)	0.49		
No. of adults	0.029 (-0.048 – 0.062)	0.80		
No. of children	0.079 (-0.043 – 0.090)	0.49		
No. of bedrooms	−0.016 (-0.070 – 0.061)	0.89		
No. of bathrooms	−0.094 (-0.138 – 0.057)	0.41		
Persons per bedroom	0.088 (-0.029 – 0.065)	0.78		
Persons per bathroom	0.085 (-0.025 – 0.055)	0.45		
Smokers	0.132 (-0.110 – 0.426)	0.25		
Pets	0.260 (0.029 – 0.328)	0.020	0.321 (0.073 – 0.368)	0.004
Household income	−0.027 (-0.102 – 0.001)	0.053	−0.274 (-0.113 to –0.014)	0.013
High/low community	0.135 (-0.064 – 0.249)	0.23		
transmission period				
Index cases characteristics				
Multiple index cases	0.183 (-0.034 – 0.361)	0.10	–	0.15
Adult/child	0.057 (-0.088 – 0.148)	0.62		
Sex	0.138 (-0.043 – 0.183)	0.22		
Hispanic/Latinx	0.245 (0.029 – 0.525)	0.029	–	0.19
Ethnicity^a^						
Comorbidities	−0.131 (-0.301 – 0.078)	0.25		
Symptomatic	−0.019 (-0.202 – 0.171)	0.87		
Duration of symptoms	0.036 (-0.008 – 0.011)	0.75		

*The slope (standardized beta coefficient) tells the strength of the effect of each independent factor on the SAR; a stronger effect is represented by a higher absolute value of the slope. In the multivariable model including household income, multiple index cases, ethnicity, and pets [F_(2, 78)_ = 6.455, p = 0.003, R = 0.381], only household income and pets significantly predicted a higher household SAR*.

a *Ethnicity of the index cases represented the predominant ethnicity in the household*.

**Table 4:** Univariate and multivariable linear regression analysis of predictors of the household attack rate (includes index cases).

**Factors**	**Univariate analysis**		**Multivariable analysis**	
	**Slope (95% CI)**	***p*-value**	**Slope (95% CI)**	***p*-value**
No. of household members	−0.182 (-0.045 – 0.002)	0.069	–	0.30
No. of adults	−0.143 (-0.058 – 0.009)	0.15		
No. of children	−0.112 (-0.059 – 0.016)	0.27		
No. of bedrooms	−0.229 (-0.080 to – 0.007)	0.021	−0.229 (-0.080 to – 0.006)	0.022
No. of bathrooms	−0.210 (-0.118 to – 0.004)	0.035		
Persons per bedroom	0.107 (-0.013 – 0.043)	0.285		
Persons per bathroom	−0.001 (-0.024 – 0.024)	0.99		
Smokers	0.108 (-0.071 – 0.242)	0.28		
Pets	−0.045 (-0.111 – 0.071)	0.66		
Household income	−0.226 (-0.066 to –0.005)	0.023	–	0.053
High/low community	0.144 (-0.025 – 0.161)	0.15		
transmission period						
Index cases characteristics				
Multiple index cases	0.220 (0.014 – 0.234)	0.027	–	0.084
Adult/child	−0.001 (-0.097 – 0.096)	0.99		
Sex	0.089 (-0.060 – 0.145)	0.42		
Hispanic/Latinx	−0.205 (-0.302 to –0.006)	0.041	–	0.070
Ethnicity						
Comorbidities	−0.135 (-0.212 – 0.042)	0.19		
Symptomatic	−0.077 (-0.146 – 0.066)	0.45		
Duration of symptoms	−0.009 (-0.007 – 0.006)	0.96		

*Because the number of bedrooms and bathrooms were highly correlated (r^2^ = 0.71, p < 0.001), the number of bathrooms was removed from the multivariable model. In the multivariate model including the number of household members, bedrooms, income, multiple index cases, and ethnicity [F_(1, 98)_ = 5.003, p = 0.028, R = 0.220], only the number of bedrooms significantly predicted the higher household attack rate (p = 0.022)*.

## Discussion

This study focused on a high predominance of low-income Hispanic/Latinx households with children in an urban setting, a vulnerable group disproportionately affected by the pandemic in the United States. We report the highest recorded mean attack rate of 83.9% within our households and SAR of 77.0% compared with previous household studies (5–14). Only a few studies have found household SAR over 50% (25, 26). All members tested positive in 60.4% of our households, showing intense clustering of SARS-CoV-2 infections within households during periods of low and high community transmission.

The number of bedrooms was a significant factor in predicting the overall household attack rate in the multivariable analysis. Fewer bedrooms likely decreased the ability to properly quarantine within the living space. Indeed, most participants reported daily face-to-face contact with a known COVID-19 positive household member, supporting decreased ability to isolate from other household members. Medical sheltering facilities or temporary housing using hotels and motels may be useful for COVID-19 positive patients for the purpose of isolation to decrease household clusters of infection (27, 28).

Lower household income was significant in the multivariable analysis to predict a higher household SAR. This is most plausibly a reflection of the combination of challenges faced by households with low socioeconomic status such as decreased access to care, an obligation to work and interact with the community, different housing patterns, neighborhood interactions, and neighborhood childcare which we did not capture in our study. Although presence of pets were also associated with higher household SAR and it seems theoretically possible that pets could briefly carry the virus and spread it throughout the household, most studies to-date do not show evidence of pet-to-human transmission (29, 30). Hispanic/Latinx ethnicity was associated with higher individual SAR. This may be due to the large Hispanic/Latinx cohort in our study. Ethnic minority populations are currently at the highest risk of infection, hospitalization, and death from COVID-19 (31, 32). Multiple data sources have shown a disproportionately high case rate amongst the Hispanic/Latinx population, with a death rate 2.3 times higher than the non-Hispanic/Latinx White population (31, 33). The higher attack rates in these communities reinforces the need for vaccination efforts specifically focused on the lower income and disproportionately affected ethnic minority populations to overcome this large disparity (34, 35).

By following our patients over the pandemic, we found that both adults and children are responsible for bringing SARS-CoV-2 to the household as the index case. A recent population-based cohort study in Canada also found that younger children were more likely to transmit SARS-CoV-2 infection compared with older children (36). During the period with lower case numbers in the community (23), we observed that the index cases were most frequently children, despite public school closures. We did not ask households about adherence to community restrictions or childcare practices during school closure, but it is possible that public health measures were not followed as strictly when children were not in school in our study population (37). A study in Spain found that children were less likely to be index cases, but higher SAR occurred when children were out of school (25). Further studies are needed to determine if children will be important drivers of transmission similar to influenza (38) during periods without community restrictions. So far, school reopenings have not been associated with a significant increase in COVID-19 community transmission (39, 40); risk must be weighed against the significant benefit of in-person schooling (41). During the largest peak of COVID-19 cases when community restrictions intensified in Los Angeles, adults 18–54 years of age comprised the highest proportion of index cases. Los Angeles County restriction orders at this time included no in-person dining, decreased capacity for essential and non-essential establishments, and no private or public gathering. Adults in that age group were the most mobile individuals within the household and at the highest risk to acquire SARS-CoV-2 from work or social contacts outside of the family.

Substantial secondary transmission occurred from both child and adult index cases in our households. Other studies have shown a significantly lower SAR when the index case is a child (16, 42); fewer studies have found a higher SAR from child index cases compared with adults (42). Almost a third of our index cases were asymptomatic overall. Of the child index cases, almost half were asymptomatic. Unlike previous studies (5, 11, 42, 43), we found that asymptomatic index cases were important transmitters of SARS-CoV-2 similarly to symptomatic index cases. Individuals who are asymptomatic or have only mild symptoms generally remain active and are thus in greater contact with others, increasing the possibility of transmission to the household and community (44, 45).

Strengths of the study include capturing and testing entire households including all children, prospectively and repeatedly performing PCR testing on NP swabs over multiple visits to identify cases, and incorporating serologic data into the analysis to catch asymptomatic individuals who may have missed the PCR positivity window. However, the findings are subject to limitations. The convenience sampling strategy, especially during the pandemic, may cause selection bias. We were unable to determine the infection order in 26 (5.5%) participants who were serologically positive but asymptomatic, which may underestimate the number of asymptomatic index cases. Misclassification of index cases is a challenge for SARS-CoV-2 transmission studies, due to the possibility of different incubation time periods and asymptomatic presentation (24). We did not perform contact tracing outside of the household to capture the source of infection outside of the household. Only half of the participants returned for convalescent serology; therefore, we may have missed individuals who developed COVID-19 immediately before enrollment or during the follow-up period and missed by the NP swab. We could not definitively differentiate second cases from subsequent cases in the households; all were included in the SAR definition. Almost all patients who were enrolled only had mild illness, and this may underestimate the SAR in households where the index patient was more severely symptomatic. A possibility exists that some of the subsequent household cases were actually acquired outside the household rather than from household transmission, the SAR did not increase during the period of increased community transmission. Viral sequencing was not performed. Cultural practices may play a role in the transmission dynamics for which we did not assess.

Households with children in low income, urban communities have an extremely high household SAR. Strategies to decrease household transmission, particularly in ethnic minority communities and low socioeconomic settings, remains crucial to controlling the SARS-CoV-2 pandemic. It is imperative to increase access to resources to make infection prevention, COVID-19 testing, and following quarantine guidelines possible and less challenging for these populations. Children, both symptomatic and asymptomatic, are important contributors to household spread. Children could be drivers of continued low level community SARS-CoV-2 circulation in vulnerable populations without vaccination. Future vaccination efforts must include special focus on children and ethnic minority populations, including the households with low income who are disproportionately affected.

## Data Availability Statement

The raw data supporting the conclusions of this article will be made available by the authors upon request, without undue reservation.

## Ethics Statement

The studies involving human participants were reviewed and approved by Children's Hospital Los Angeles Institutional Review Board. Written informed consent to participate in this study was provided by the participants' legal guardian/next of kin.

## Author Contributions

PP conceptualized and designed the study, analyzed the data, and reviewed and revised the manuscript. MT and CM collected data, carried out the initial analyses, and drafted the initial manuscript. SM helped carry out the initial analysis and draft the initial manuscript. LT and AP designed the data collection instruments, collected data, and reviewed and revised the manuscript. JN helped design the data collection instruments and reviewed and revised the manuscript. YL, PT, and AG helped design the study and reviewed and revised the manuscript. JJ and SP collected data and reviewed and revised the manuscript. EA and JD reviewed and revised the manuscript. All authors approved the final manuscript as submitted and agree to be accountable for all aspects of the work.

## Funding

This work was supported by the National Institute of Allergy and Infectious Diseases at the National Institutes of Health [U01AI144616-02S1] and a grant from Open Philanthropy.

## Conflict of Interest

PP has received consultant fees from Sanofi-Pasteur and Seqirus and also receives research funding from AstraZeneca and Pfizer for unrelated studies. AG has received consultant fees from Janssen. The remaining authors declare that the research was conducted in the absence of any commercial or financial relationships that could be construed as a potential conflict of interest.

## Publisher's Note

All claims expressed in this article are solely those of the authors and do not necessarily represent those of their affiliated organizations, or those of the publisher, the editors and the reviewers. Any product that may be evaluated in this article, or claim that may be made by its manufacturer, is not guaranteed or endorsed by the publisher.

## Abbreviations

CHLA: Children's Hospital Los Angeles
CI: Confidence Interval
CT: Cycle threshold
COVID-19: Coronavirus disease 2019
ELISA: Enzyme-linked immunosorbent assay
FDA: Food and Drug Administration
IQR: Interquartile range
NP: Nasopharyngeal
RBD: Receptor binding domain
RNP: RnaseP
RT-PCR: Reverse Transcriptase-Polymerase Chain Reaction
SAR: Secondary attack rate
SARS-CoV-2: Severe acute respiratory syndrome coronavirus-2
SD: Standard deviations.
