# Supplementary material for: SARS-CoV-2 Transmission Dynamics in Households With Children, Los Angeles, California
Source: Front Pediatr. 2022 Jan 5;9:752993. doi: 10.3389/fped.2021.752993 (PMC8767010; doi:10.3389/fped.2021.752993)
Supplement: Supplementary file 1 [file Table_1.pdf]

Supplementary - SARS-CoV-2 Transmission Dynamics in Households with Children, Los Angeles, California

Supplemental Table 1. Laboratory-confirmed index cases per time period, according to age group. Children index cases were more frequent during periods of lower COVID-19 community transmission compared to the period of very high community transmission levels. (p=0.018 if compare all 3 periods, p=0.006 if compare Period 1 and 2 vs. 3). The periods were determined by the epidemic curve shown in Figure 1.

**Age group (years)**	**Period 1 June (start of study) to August 31, 2020**	**Period 2 September 1 to October 31, 2020**	**Period 3 November 1 to December 31, 2020**
<12	16 (36.4)	8 (21.1)	14 (25.9)
12-17	9 (20.5)	2 (13.3)	4 (7.4)
18-29	4 (9.1)	1 (6.7)	16 (29.6)
30-54	12 (27.3)	4 (26.7)	18 (33.3)
≥55	3 (6.8)	0 (0)	2 (3.7)
Total	44 (38.9)	15 (13.3)	54 (47.8)

Supplemental Table 2. Sensitivity analysis of the household secondary attack rate (SAR) after reclassification of cases with onset within 1 to 5 days of the original index case within the household to account for the possibility of misclassification of index cases.

	**Households with known infection order based on symptoms and PCR testing**	**Reclassify cases within 1 day of original index as index**	**Reclassify cases within 2 days of original index as index**	**Reclassify cases within 3 days of original index as index**	**Reclassify cases within 4 days of original index as index**	**Reclassify cases within 5 days of original index as index**
**# Households**	83	83	83	83	83	83
**# Index cases**	113	143	165	184	201	219
**Household SAR, % (95% CI)**	77.0 (69.4-84.6)	74.5 (66.3-82.7)	71.1 (61.9-80.4)	68.4 (58.6-78.3)	65.9 (55.3-76.5)	63.7 (52.7-74.6)
